# Vieillir avec le VIH au Sénégal, perspectives anthropologiques pour une politique de santé publique

**DOI:** 10.48327/mtsi.v6i1.2026.769

**Published:** 2026-02-27

**Authors:** Bernard TAVERNE, Gabrièle LABORDE-BALEN, Seynabou DIOP, Catherine FALL, Marcel Ndiana NDIAYE, Pierre Thiouthy SARR, Khoudia SOW

**Affiliations:** 1 TransVIHMI (Université de Montpellier, INSERM, Institut de recherche pour le développement), 911, avenue Agropolis - BP 64501 - 34394 Montpellier Cedex 5, France; 2 Centre régional de recherche et de formation à la prise en charge clinique de Fann (CRCF), CNHU de Fann, Dakar, Sénégal

**Keywords:** VIH, Personnes âgées, ARV, Stigmatisation, Dépendance, Comorbidité, Vieillissement, Milieu urbain, CHNU de Fann, Centre de santé Dominique, Région de Dakar, Sénégal, Afrique subsaharienne, HIV, Older adults, ARV, Stigmatization, Dependency, Comorbidity, Aging, Urban environment, Fann University Hospital Center, Dominique Health Center, Dakar Region, Senegal, Sub-Saharan Africa

## Abstract

**Introduction:**

En Afrique, grâce à l’efficacité des antirétroviraux (ARV), de plus en plus de personnes vieillissent avec le VIH. Au Sénégal, en 2022, 34 % des personnes vivant avec le VIH (PVVIH) et recevant des traitements ARV étaient âgées de 50 ans ou plus. Le vieillissement s’accompagne d’une évolution progressive des besoins médicaux, psychologiques et sociaux des PVVIH, qui s’accentuent avec l’âge. Cet article présente les résultats d’une étude anthropologique visant à décrire et analyser les conditions de vie et de prise en charge des PVVIH âgées de 70 ans et plus, en lien avec le contexte sanitaire et social en milieu urbain.

**Matériel et méthode:**

L’étude ethnographique a été menée entre 2022 et 2023 dans la région métropolitaine de Dakar. Les entretiens et observations ont porté sur 31 personnes âgées vivant avec le VIH (PAVVIH), dont 14 femmes, âgées en moyenne de 72 ans (au plus 90 ans). La durée médiane du traitement ARV était de 16 ans (durée maximale : 22 ans). Ont également été interrogés six aidants familiaux, quatre médiateurs associatifs, une infirmière, cinq médecins dont un gériatre, ainsi que les principaux responsables nationaux de la lutte contre le sida.

**Résultats:**

Les PAVVIH rencontrées témoignent d’une forme d’habituation face au VIH, et font preuve d’une très bonne adhésion au traitement ARV. Cependant, leur quotidien demeure marqué par la stigmatisation sociale persistante du VIH qui conduit à diverses formes d’auto-exclusion sociale et de stratégies de maintien du secret autour de leur maladie. Sur le plan médical, toutes sont en suppression virologique, mais elles sont confrontées à de multiples comorbidités et incapacités fonctionnelles qui induisent des dépenses de santé croissantes. La précarité économique et l’insuffisance des dispositifs de protection sociale entraînent une dépendance accrue à l’égard de leurs proches. Les familles sont les principales pourvoyeuses des soins. L’attention portée aux aînés est cependant déterminée par la nature et la qualité des liens tissés tout au long de la vie, dans une forme d’héritage des relations familiales.

**Conclusion:**

Cette recherche s’inscrit dans l’apport de l’anthropologie à l’analyse et la compréhension des systèmes de soins et de leur capacité à prendre en charge des populations spécifiques. Dans une perspective de santé publique, cette analyse plaide pour une prise en compte des spécificités des besoins sanitaires et sociaux des PAVVIH par les structures sanitaires, les acteurs communautaires et les autorités de santé afin de permettre un vieillissement digne des PAVVIH.

## Abstract (en)

**Introduction:**

Thanks to the effectiveness of antiretroviral treatment (ART), more and more people in Africa are aging with HIV. In Senegal, 34% of people living with HIV (PLHIV) who received ART were 50 years of age or older in 2022. As people age, their medical, psychological, and social needs gradually change and increase. This article presents the results of an anthropological study that aimed to describe and analyze the living conditions and care of PLHIV aged 70 and over in urban areas, in relation to the health and social context.

**Materials and methods:**

The ethnographic study was conducted from 2022 to 2023 in the Dakar metropolitan area. The study focused on 31 older people living with HIV (OPLHIV), including 14 women, with an average age of 72 (maximum age 90). The median duration of ART was 16 years (maximum: 22 years). Six family caregivers, four community mediators, one nurse, and five doctors (including a geriatrician) were also interviewed, as well as the main national officials responsible for the fight against AIDS.

**Results:**

The OPLHIV who were interviewed reported habituation to HIV and demonstrated excellent adherence to ART. However, persistent social stigma surrounding HIV continues to affect their daily lives, leading to various forms of self-exclusion and strategies to keep their illness secret. Medically, they are all in virological remission; however, they face multiple comorbidities and functional disabilities that lead to increased healthcare costs. Economic insecurity and inadequate social protection systems lead to increased dependence on loved ones. Families are the main providers of care. However, the attention given to the elderly depends on the nature and quality of the relationships they have built throughout their lives.

**Conclusion:**

This research contributes to anthropology’s analysis of healthcare systems and their capacity to care for specific populations. From a public health perspective, the findings of this analysis call for health facilities, community stakeholders, and health authorities to consider the unique health and social needs of OPLHIV to enable them to age with dignity.

## Introduction

### Contexte international

L’efficacité biologique des médicaments antirétro-viraux (ARV) a eu un impact majeur sur la vie des personnes vivant avec le VIH en transformant le pronostic d’une maladie fatale à brève échéance en une maladie chronique. En quelques années, l’espérance de vie des personnes vivant avec le VIH (PVVIH) s’est rapprochée de celle de la population générale [32,50]. Cette évolution a conduit à un vieillissement progressif des cohortes de patients, y compris en Afrique [1,34]. Dès 2013, l’ONUSIDA a signalé l’augmentation du nombre de personnes âgées de 50 ans et plus vivant avec le VIH (PAVVIH), attribuée au succès des ARV, à la baisse de l’incidence chez les jeunes et à la persistance de comportements à risque chez les personnes âgées [39]. En 2022, conformément à ces projections, les PAVVIH représentaient plus de 50 % des PVVIH dans les pays à revenu élevé et 30 à 35 % en Afrique subsaharienne [15]. En 2011, on estimait à 4 millions le nombre de PAVVIH dans ces régions; ce nombre devrait être d’environ 9 millions en 2040 [26].

L’ONUSIDA et divers auteurs ont recommandé des politiques nouvelles de prévention, dépistage et traitement, y compris pour les maladies non transmissibles, adaptées aux PAVVIH [1,39]. Néanmoins les pays d’Afrique de l’Ouest et du Centre tardent encore à les mettre en œuvre [22]. Par ailleurs, le vieillissement des PVVIH s’inscrit dans une dynamique démographique plus large : en Afrique subsaharienne, la population âgée de 65 ans et plus devrait tripler entre 2022 et 2050, passant de 34 à 98 millions [33]. Cette évolution accentuera la pression sur les systèmes de santé [48], actuellement peu préparés. En l’absence de politiques solides en faveur des personnes âgées [28], le soutien aux aînés repose actuellement surtout sur les familles dont une grande partie vit dans des situations de pauvreté chronique [20]. Les conditions de vie des personnes âgées restent donc précaires et leur accès aux soins souvent limité. Pourtant, selon l’OMS, le processus du vieillissement dans la dignité suppose de conserver autonomie, respect et qualité de vie décente, d’avoir accès à des soins appropriés et de bénéficier d’un soutien émotionnel et d’une reconnaissance sociale [40], un idéal encore loin d’être atteint en Afrique subsaharienne.

### Contexte au Sénégal

Le Sénégal fut le premier pays d’Afrique francophone à mettre en place un programme gouvernemental d’accès aux médicaments ARV en 1998 [10]. Depuis 2003, ARV et charge virale sont gratuits, mais une partie des coûts des soins reste à la charge des patients. L’épidémie de VIH au Sénégal est de type concentré, avec une prévalence faible dans la population générale (0,3 %) et une prévalence élevée dans certaines localités et chez les populations les plus vulnérables. En 2022, 34 % des 32 000 personnes recevant des ARV étaient âgées d’au moins 50 ans [43].

Sur le plan socio-économique, environ 38 % de la population sénégalaise et la moitié des ménages dirigés par des personnes âgées vivent sous le seuil de pauvreté [44]. Les politiques sociales sont limitées : seules 20 % des personnes âgées perçoivent une pension de retraite. Le principal soutien institutionnel pour les personnes âgées, le Plan Sésame, créé en 2006, vise à fournir des soins gratuits aux plus de 60 ans sans autre couverture médicale, mais son application reste inégale, freinée par des retards de remboursement de l’État aux structures de santé. En pratique, les personnes âgées supportent une large part des dépenses de santé [49]. Le soutien familial reste leur principale source d’appui, d’autant qu’il est ancré dans l’éthique sociale de solidarité intergénérationnelle [12].

Au Sénégal, la cohabitation intergénérationnelle est fréquente [44], elle favorise l’inclusion mais met à l’épreuve la solidarité, limitée par l’instabilité économique des ménages. Dans un contexte de polygamie, les inégalités de genre sont marquées : près de la moitié des femmes de plus de 70 ans sont veuves *versus* 3 % des hommes et leur situation est souvent précaire [19]. Dans une société majoritairement musulmane (95 %), les normes sociales valorisent la vieillesse et encouragent le sentiment religieux chez les personnes âgées [3,18]. Malgré cette reconnaissance sociale, leurs conditions de vie restent difficiles [23,36,37]. Le VIH demeure une maladie stigmatisante, d’autant que la faible prévalence rend peu visibles les PVVIH [45]. Dans ce contexte, vieillir avec le VIH constitue un défi majeur.

### Une recherche anthropologique au Sénégal

Depuis les années 2000, une abondante littérature anthropologique a documenté les conditions sociales, culturelles et politiques affectant l’accès aux ARV sur le continent africain [13,35,46] et l’impact de ces traitements sur les PVVIH [11,54]. Une riche littérature sur le vieillissement et les relations de soins en Afrique existe également [17,18,28,31]. Néanmoins, les études anthropologiques sur le vieillissement avec le VIH en Afrique francophone sont encore rares et des questions demeurent : comment le vieillissement et l’infection par le VIH interagissent-ils dans le contexte des sociétés africaines ? Quelles sont les expériences et les perceptions des PAVVIH et de leurs familles face au vieillissement et aux situations de dépendance ? Quelles sont les réponses sanitaires et sociales apportées par les proches et les institutions ?

Dans le cadre du projet *Grand Âge et VIH, anthropologie de la maladie et du vieillissement*, financé par Sidaction, une étude a été menée à Dakar pour analyser les conditions de vie et les relations de soins des PAVVIH.

## Méthodes

### Dates et sites de l’étude

Les enquêtes ont été réalisées entre juin 2022 et avril 2023, dans trois structures de santé : le Centre de traitement ambulatoire (CTA) et le Centre de recherche et de formation à la prise en charge clinique de Fann (CRCF), tous deux situés dans le Centre hospitalier national universitaire de Fann à Dakar, ainsi que le Centre de santé Baye Talla Diop, également connu sous le nom de « Dominique », dans la banlieue dakaroise. Le CTA et le CRCF, créés respectivement en 1995 et 2005 sont des structures de référence au niveau national et international dédiées à la prise en charge médicale et sociale des PVVIH. Elles sont impliquées dans la formation sur le VIH de la plupart des acteurs de santé du pays. Environ 3 000 PVVIH y sont suivies, parmi lesquelles près 50 % sont âgées de plus de 50 ans. Le troisième site est une structure médicale urbaine polyvalente dans laquelle sont suivies environ 450 PVVIH. Dans chacun de ces sites, des médiateurs – membres des associations de soutien aux PVVIH – participent à la prise en charge psychosociale des PVVIH.

### Population de l’étude

L’étude a concerné des personnes vivant avec le VIH âgées d’au moins 70 ans, âge auquel la survenue de comorbidités et d’incapacités fonctionnelles entraînent une réduction de l’autonomie et l’émergence de situations de dépendance [16]. Les participants ont été sélectionnés sur la base d’un choix raisonné, en veillant à diversifier les profils selon le sexe, l’état civil, l’orientation sexuelle, la situation économique et le site de prise en charge. Le nombre d’entretiens a été défini sur la base du principe de saturation de l’information [25]. Au total, 31 PAVVIH ont été incluses (14 femmes et 17 hommes), avec un âge médian de 73 ans (70-90 ans). Pour la moitié d’entre elles, le diagnostic de VIH avait été posé avant 2005; le plus ancien diagnostic remontait à 1987. La durée médiane de la thérapie antirétrovirale était de 16 ans (6-22 ans) et 8 personnes étaient traitées depuis plus de 20 ans.

Des entretiens ont été également réalisés avec cinq médecins, dont un gériatre, un infirmier, quatre médiateurs communautaires, et six aidants familiaux. Par ailleurs, des entretiens informels ont été conduits avec les principaux responsables nationaux de la lutte contre le sida, ainsi qu’avec divers acteurs communautaires : associations de PVVIH, associations engagées dans la prise en charge d’autres maladies (AVC, diabète, hépatites) et la principale organisation de personnes âgées du pays, le Conseil national des aînés du Sénégal. Au total, 51 personnes ont été interrogées.

### Méthode de collecte des données

Les informations ont été recueillies au moyen d’entretiens semi-structurés et d’observations directes. L’étude a été menée par : (i) deux assistants de recherche sénégalais, tous deux titulaires d’un master 2, en santé communautaire pour l’une et en sociologie pour l’autre; (ii) une médiatrice communautaire membre d’une association de femmes vivant avec le VIH et travaillant depuis près de 25 ans auprès des PVVIH à Dakar; et (iii) trois anthropologues seniors – dont deux sont également médecins – ayant entre 15 et 30 ans d’expérience dans la recherche sur le VIH en Afrique de l’Ouest.

La plupart des 26 entretiens avec les PAVVIH a été réalisée dans les sites de prise en charge à leur demande, principalement pour des raisons de confidentialité; cinq entretiens ont toutefois eu lieu au domicile, ce qui a permis d’observer les conditions de vie des enquêtés. Une allocation de transport a été remise aux participants lorsque l’entretien se déroulait dans une structure de santé, tandis que de petits présents (notamment alimentaires) ont été offerts lors des visites à domicile. Dans certaines situations d’extrême pauvreté, un soutien ponctuel a pu être apporté, par exemple pour l’achat de médicaments.

Les entretiens ont été menés à l’aide d’un guide thématique (voir Annexe) pour chaque catégorie d’interlocuteurs, qui a notamment exploré : l’histoire de vie de la personne et l’histoire de la maladie, son itinéraire thérapeutique, son expérience de la maladie et celle des membres de sa famille, les relations intrafamiliales, les réseaux de solidarité et l’organisation des soins. Les thèmes ont été développés et affinés au fil du temps en fonction des sujets abordés par les personnes interrogées ou des questions soulevées lors des séances d’analyse des récits.

À l›issue des entretiens avec les patients, et avec leur accord, les informations obtenues ont été complétées par celles des intervenants sociaux et médicaux, en particulier les informations biocliniques extraites des dossiers médicaux. Les entretiens avec les professionnels de santé ont été menés en français, tandis que tous les autres ont été menés dans la langue locale (wolof), traduits et transcrits en français. L’approbation éthique de l’étude a été obtenue auprès du Comité national d’éthique pour la recherche en santé du Sénégal. Le protocole a été expliqué à chaque participant, qui a reçu des informations détaillées sur l’étude et a eu la possibilité de poser des questions avant de donner son consentement oral. La participation aux entretiens était entièrement volontaire. Tous les membres de l’équipe, formés à l’éthique de la recherche, ont signé des accords de confidentialité. Lors de la transcription, chaque participant s’est vu attribuer un pseudonyme afin de garantir l’anonymat. Tous les prénoms mentionnés dans les citations et les fragments de récits de vie dans cet article sont fictifs.

### Cadre théorique

Notre approche théorique s’appuie sur trois concepts clés : le premier est celui de « transition » tel que défini par Grenier [21], dans une perspective de parcours de vie. Ce concept met en évidence le caractère fluctuant, évolutif et singulier des « transitions » qui jalonnent l’existence. Le deuxième est la perspective intersectionnelle inspirée de Crenshaw [6] qui permet de décrire l’imbrication et l’interdépendance des multiples relations sociales inégalitaires dans lesquelles les individus sont pris. Le troisième concept est celui de « l’éthique du *care* », que Tronto définit comme un ensemble de valeurs morales et de pratiques visant à soutenir les personnes qui ne peuvent pas vivre de manière autonome [51]. Ce concept distingue quatre étapes de ce processus : reconnaître le besoin d’autrui (*care about*), assumer sa responsabilité d’y répondre (*taking care of* ), prodiguer effectivement les soins (*care giving*) et recevoir ces soins (*care receiving*).

Cette approche permet d’analyser à quel moment et de quelle manière les soins sont dispensés, ainsi que leur contribution au bien-être de la personne [52]; elle éclaire les pratiques du *care* mises en œuvre par les différents acteurs familiaux, communautaires et institutionnels dans l’accompagnement des PAVVIH.

Ces trois concepts – transition de parcours de vie, intersectionnalité et éthique du *care* – sont étroitement articulés. Cette interconnexion est essentielle pour comprendre comment les déterminants biologiques et sociaux influencent de manière diverse l›expérience du vieillissement avec le VIH.

### Méthode d’analyse

Le corpus de données (entretiens et observations) a fait l’objet d’une analyse inductive de contenu, inspirée de la *grounded theory*. Cette approche qualitative vise à faire émerger les catégories d’analyses directement à partir des données empiriques. Le codage manuel des données a été réalisé de manière itérative au fur et à mesure de l’avancement de l’étude, permettant l’identification de nouveaux thèmes en complément de ceux déjà identifiés [38]. Inspirés par la « *transition in life course* » proposée par Grenier [21], nous avons développé une démarche biographique destinée à reconstituer, pour chaque individu, un récit de vie situant la personne dans son parcours historique – de la naissance à aujourd’hui – dans les sphères familiale, professionnelle et médicale. L’approche biographique permet d’analyser la manière dont chacun interprète subjectivement son existence et ses relations avec les membres de sa famille et de son entourage. Elle offre ainsi la possibilité de saisir le social dans sa forme individuelle – les événements-clés de la vie, façonnés par des dynamiques collectives (éducation, travail, parentalité, etc.) – tout en le replaçant dans le contexte social global, marqué par des conditions culturelles, économiques et politiques plus larges (cadre politique et historique, normes et valeurs collectives, etc.) [7].

Les analyses ont été menées par les anthropologues seniors, conjointement avec les assistants de recherche dans une perspective pédagogique. Les résultats préliminaires ont ensuite été discutés avec des membres d’associations communautaires de PVVIH (dont la plupart étaient eux-mêmes séropositifs) afin de renforcer leur participation au processus de recherche.

## Résultats

### Le VIH et les ARV, un vécu apaisé

Toutes les PAVVIH rencontrées décrivent une forme d’habituation à leur maladie :

« *On vit avec; ce n’est plus un problème, on s’y habitue. On oublie presque qu’on est malade* » (Aminata, 70 ans, 21 ans sous ARV).

La plupart de ces personnes ont été diagnostiquées à un stade avancé de la maladie alors qu’elles souffraient déjà de diverses infections opportunistes. Pour d’autres, le VIH n’a été diagnostiqué qu’à l’occasion de symptômes qu’elles considéraient comme mineurs, tels que fatigue, toux ou fièvre. Dans tous les cas, l’annonce a été vécue comme une tragédie. Plusieurs personnes se souviennent de la conviction partagée avec leurs proches qu’elles allaient mourir rapidement. Toutes évoquent également la détresse liée à la stigmatisation, le VIH étant perçu comme une maladie touchant les hommes homosexuels et les travailleuses du sexe.

Amadou a 70 ans. Ancien mécanicien sur des bateaux de pêche, il a sillonné les côtes de l’Afrique de l’Ouest. À 55 ans, il est hospitalisé pour un abcès au poumon; sa séropositivité est alors découverte.

« *À l›époque, j›ai cru que ma vie était finie* » ditil. Après 15 ans de traitement ARV, en 2022, au moment de l›entretien, il déclare : « *Pour moi, le VIH n’est pas une maladie; une maladie, c’est quelque chose qui vous cloue au lit; le VIH ne m’empêche pas de faire ce que je veux; cela ne me fatigue pas. Je prends juste mes médicaments* » (Amadou, 70 ans, 15 ans sous ARV).

Comme Amadou, toutes les personnes interrogées expriment le sentiment d’avoir surmonté l’épreuve. Grâce au traitement ARV, leur état de santé a connu une amélioration spectaculaire. La disparition des stigmates les plus visibles liés à la maladie (notamment la maigreur extrême et la diarrhée) a rendu possible le retour à une certaine normalité tant sur le plan physique que social. Toutes les personnes ont témoigné de l’aide reçue de la part des médiateurs communautaires et des associations.

Les difficultés liées aux premiers traitements appartiennent désormais au passé. Au début des années 2000, les schémas thérapeutiques anti-rétroviraux pouvaient atteindre une vingtaine de comprimés par jour et entrainer des effets secondaires épuisants : nausées, diarrhées, insomnies. Grâce aux progrès médicaux, en 2025, ces traitements se limitent le plus souvent à un seul comprimé par jour et présentent moins d’effets secondaires.

Toutes les PAVVIH interrogées insistent sur leur observance exemplaire et l’importance qu’elles accordent à leur traitement ARV, ce que les professionnels de santé ont confirmé. Elles affirment avoir retrouvé une vie « normale ». Celles qui ont bénéficié des premiers traitements dans les années 2000 se considèrent comme de véritables survivantes.

### Silence, secret, partage

Si les PAVVIH de l’enquête disent avoir retrouvé une forme de normalité sociale, leurs conditions de vie restent marquées par la peur de la stigmatisation sociale liée au VIH.

« *Le VIH est une maladie qu’il ne faut pas dévoiler, une maladie qui n’est pas belle à voir* » (Ndeye, 74 ans, dont 16 ans sous ARV).

Les circonstances du diagnostic ont souvent déterminé les conditions de l’annonce. Ainsi, lorsqu’une personne était hospitalisée dans un état grave, il arrivait fréquemment que les professionnels de santé révèlent le diagnostic à une personne de l’entourage immédiat sans en référer à l’intéressée. Toutefois, dans la plupart des cas, le malade a été directement informé et encouragé à partager le diagnostic avec un proche susceptible d’apporter un soutien matériel ou moral. Les confidents étaient généralement le conjoint, la personne qui les accompagnait aux consultations ou encore celle qui finançait les soins. Souvent, les femmes préféraient informer une mère ou une sœur. Les veuves étaient plus enclines à partager l’information avec leurs enfants adultes, persuadées d’avoir contracté le VIH avec leur défunt mari et soucieuses de ne pas être accusées d’inconduite. La crainte d’un jugement moral sur les circonstances de l’infection demeure le facteur central qui oriente le choix des personnes informées et le moment de la révélation. Les hommes interrogés ont divulgué plus rarement leur statut sérologique, parfois à leur épouse, à une sœur ou à un frère. Le partage au sein du couple était plus fréquent au moment du diagnostic que lors des remariages ultérieurs. Avec le temps, la parole s’est raréfiée. Dix ou vingt ans après le diagnostic, certaines PVVIH gardent encore un silence absolu sur leur maladie.

Chez les hommes ayant des rapports sexuels avec des hommes (HSH), la réticence est encore plus forte. Paul (70 ans) a longtemps caché son homosexualité. Marié pour éviter les soupçons, il est devenu père d’un fils, puis a divorcé après le scandale familial provoqué par la découverte de son orientation sexuelle. Rejeté par sa famille, il a quitté le Sénégal avant d’y revenir en 2010, moment où il a été diagnostiqué positif au VIH. Il a décidé de taire sa maladie, en particulier à son fils.

« *J’ai tout fait pour lui, tout. Mais je me dis qu’à ses yeux, je suis une honte* » (Paul, 70 ans, 14 ans sous ARV).

Karim, âgé de 84 ans, mécanicien à la retraite, illustre une autre forme de silence. Vivant avec sa femme, 2 de ses enfants et ses petits-enfants, il n’a jamais parlé ouvertement de sa maladie à ses enfants afin de préserver sa dignité :

« *Il y a des conversations que je n’aurai jamais avec un enfant. Je n’ai jamais parlé de ma maladie avec eux; ils le savent, car en 2000, ma fille aînée m’a accompagné à l’hôpital. Je sais qu’ils sont au courant, mais je ne leur en ai jamais parlé* ». (Karim, 84 ans, 22 ans sous ARV).

Le tabou entourant la sexualité des personnes âgées accentue les réticences chez les personnes dépistées à un âge avancé. Les femmes âgées sont souvent veuves, ayant perdu leur conjoint à cause de la maladie, ou en raison de la différence d’âge dans le contexte de polygamie. Toutes ont subi des pressions familiales pour se remarier, mais aucune n’a accepté, craignant que leur nouveau conjoint ne révèle leur maladie.

L’apparition de handicaps liés à l’âge (cécité, réduction de la mobilité) introduit toutefois de nouveaux dilemmes. La dépendance vis-à-vis de l’entourage pour les activités quotidiennes (comme la prise de médicaments ou le déplacement pour les visites médicales) oblige parfois à réévaluer les choix initiaux et à lever le secret. Cette révélation peut clarifier un non-dit et susciter de la compassion. Dans d’autres cas, cela ravive des conflits passés et des accusations de dissimulation.

### La survenue des comorbidités

Les personnes de l’étude expriment un rapport désormais apaisé avec le VIH. Toutes maintiennent depuis plusieurs années une charge virale indétectable. Elles soulignent l’importance qu’elles accordent à la prise régulière du traitement ARV et au suivi biologique.

Avec l’âge, les préoccupations se déplacent : ce ne sont plus tant le VIH et son traitement qui inquiètent mais l’apparition d’autres maladies chroniques et des handicaps fonctionnels. Les plus fréquents sont l’hypertension artérielle, le diabète et ses complications (accident vasculaire cérébral, insuffisance rénale, maladies cardiaques et cécité) et les douleurs ostéoarticulaires. Ces maladies compliquent le suivi médical, imposent de nouveaux parcours de soins, nécessitant la consultation de spécialistes hors des structures habituelles de suivi du VIH. L’une des principales sources d’inquiétude concerne la capacité à financer des examens complémentaires et des médicaments pour ces affections chroniques.

« *Je souffre d’hypertension artérielle. Le médecin m’a prescrit un médicament. Je l’achetais à la fin de chaque mois. Mais il est trop cher pour moi. J’ai du mal à trouver la somme nécessaire pour l’acheter. J’ai donc été obligée d’arrêter de le prendre par manque de moyens* ». (Aminata, 70 ans, 22 ans de TARV).

Les coûts cumulés représentent rapidement des sommes importantes, difficiles à mobiliser sur le long terme. Pour les personnes polypathologiques, la prise de médicaments supplémentaires complique la vie quotidienne. Par habitude et conviction, certaines privilégient leurs ARV au détriment d’autres médicaments jugés moins prioritaires.

### Déclin économique et précarité financière

Les récits de vie recueillis montrent que l’infection à VIH a constitué un bouleversement dans la vie des individus. La maladie a entrainé diverses formes de ruptures : perte d’emploi, divorce, veuvage, conflits familiaux. Les soins médicaux et le traitement ont permis un retour progressif à une relative stabilité et les personnes ont pu se réinvestir dans leurs rôles sociaux. Avec l’avancée en âge, cet équilibre se fragilise à nouveau. La réduction puis l’arrêt des activités professionnelles (qu’il s’agisse du départ à la retraite pour les salariés ou de l’apparition d’incapacités fonctionnelles) marquent une nouvelle rupture biographique.

Pour les personnes issues du secteur formel, le montant des pensions de retraite reste modeste. Le montant le plus élevé rapporté dans l’étude est de 35 000 FCFA/mois (53 €). Avec les régimes de réversion, les veuves perçoivent des montants encore plus bas (25 000 FCFA/mois, soit 38 €), surtout dans les foyers polygames où la pension est divisée entre les épouses.

La majorité des personnes avaient travaillé dans le secteur informel. Sans pension ni revenus réguliers, certaines ont vendu leurs biens pour couvrir leurs dépenses de santé; d’autres ont été contraintes à des déménagements successifs, qui les poussent progressivement vers la périphérie urbaine à la recherche de loyers plus abordables. La plupart des PAVVIH rencontrées ont déclaré avoir cherché à travailler aussi longtemps que leur condition physique le leur permettait, comme l’explique Mohamed.

« *J’ai travaillé pendant des années dans une usine de poisson, je déchargeais des caisses, mais j’ai dû arrêter il y a trois ans, c’était trop dur* ». (Mohamed, 74 ans, 19 ans sous ARV).

### Qui prend en charge les PAVVIH ?

Les résultats de l’étude montrent que les PAVVIH sont confrontées à diverses formes de dépendance qui s’étendent progressivement à tous les domaines de la vie quotidienne, les obligeant à mobiliser différents types de soutien, principalement de trois types : les structures de santé, les associations de PVVIH et les familles.

### Les structures de santé

Dans le cadre du suivi de l’infection à VIH, les visites semestrielles dans les structures de prise en charge rythment la vie des PAVVIH rencontrées. Elles permettent le renouvellement des ordonnances d’ARV et la réalisation des analyses biologiques. Ces rendez-vous sont intégrés dans la vie quotidienne et ces lieux de soins perçus comme des espaces de confiance et de reconnaissance. Les enquêtés s’y disent respectés et bien accueillis : au fil des ans, des liens personnels se sont tissés avec les professionnels de santé qui les appellent par des surnoms familiers et respectueux (« Tonton Faye » ou « Tanti Fanta »). Ces relations offrent parfois des privilèges : éviter les files d’attente, bénéficier de la gratuité de certains examens ou recevoir des dons de nourriture. Ces patients sont parfois invités à participer à la célébration d’événements nationaux organisés dans les structures de soins (Journée mondiale de lutte contre le sida, Octobre Rose…), où elles sont mises à l’honneur avec une affection réelle, teintée parfois d’une pointe de condescendance. Leur présence incarne aux yeux des soignants un témoignage vivant de la qualité des soins et du succès de la prise en charge.

Même lorsque les trajets sont longs et coûteux, les PAVVIH n’envisagent pas d’être suivies ailleurs. Fatou, suivie depuis 20 ans dans un hôpital de référence de Dakar, illustre cet attachement. Bien que des centres de santé plus proches de chez elle fournissent des ARV, elle insiste pour venir à l’hôpital :

« *Je préfère venir ici car ils sont spécialisés* [dans le VIH] *et je n’ai pas peur par rapport à la confidentialité ici, contrairement au centre de santé. On ne parle de notre maladie qu’ici; c’est le seul endroit où on en parle et où on voit nos pairs* » (Fatou, 70 ans, 20 ans sous ARV).

Les soignants de ces structures adoptent une attitude protectrice et se considèrent souvent comme une « seconde famille » pour leurs patients qu’ils suivent depuis des années.

En revanche, lorsque d’autres maladies chroniques comme l’hypertension ou le diabète imposent une orientation vers des services spécialisées, les personnes vivent une expérience contrastée : anonymes dans les files d’attente interminables, confrontées aux coûts élevés des consultations, des examens complémentaires et des médicaments, elles peinent à trouver la même qualité relationnelle. La peur de la stigmatisation les conduit parfois à taire leur infection à VIH, accentuant leur sentiment de vulnérabilité.

### Les associations de PVVIH

Depuis les années 2000, les associations de PVVIH jouent un rôle central dans la prévention, le conseil, le dépistage et l’accompagnement des patients. Certains de leurs membres interviennent comme médiateurs au sein des structures de santé. Lors de nos entretiens, les responsables de ces associations ont indiqué être conscients des difficultés croissantes rencontrées par leurs membres vieillissants. Pourtant, aucun dispositif spécifique n’a été mis en place à leur intention. Ce n’est que depuis l’épidémie de Covid en 2020 que des initiatives ciblées ont émergé. La première a été la livraison à domicile des ARV afin de pallier les restrictions de déplacement imposées par les mesures sanitaires. Depuis, ce service s’est maintenu et plusieurs organisations le proposent aux personnes âgées qui éprouvent des difficultés à se rendre dans les structures de soins pour se procurer leurs ARV.

### Le soutien familial

Au sein des familles, la prise en charge quotidienne des PAVVIH repose sur différents membres du foyer. Conformément à la répartition genrée des tâches ménagères, ce sont le plus souvent les femmes (filles, belles-filles et nièces) qui assurent les soins aux personnes âgées dépendantes. D’autres membres de la famille, résidant à proximité ou vivant à l’étranger, peuvent également être sollicités, notamment pour un appui financier.

Les témoignages des PAVVIH de notre étude montrent des configurations de soutien familial variables selon le genre et la situation socio-économique. Chez les hommes mariés âgés vivant avec le VIH, les épouses apparaissent comme les principales pourvoyeuses de soins au quotidien. Elles sont souvent beaucoup plus jeunes, informées ou non de la maladie de leur conjoint. Certaines vivent elles-mêmes avec le VIH. Quel que soit leur âge, les hommes conservent généralement le statut de chef de famille dès lors qu’ils perçoivent une pension de retraite ou disposent de revenus réguliers. Dans un contexte marqué par un taux important de chômage chez les jeunes, cette pension devient une ressource essentielle pour le foyer. Elle est fréquemment mobilisée au bénéfice de l’ensemble de la famille, parfois au détriment des besoins propres des aînés.

Ousseynou, ancien instituteur, vivant au domicile familial, est le seul à bénéficier d’un revenu régulier :

« *C’est moi qui assure les charges du ménage; quand la retraite arrive, je paie les factures d’eau et d’électricité, ainsi que toutes les dépenses quotidiennes, car les enfants ne travaillent pas. Ma retraite ne suffit pas, alors je me débrouille* » (Ousseynou, 81 ans, 11 ans sous ARV, 10 personnes à charge).

Néanmoins, certains hommes âgés se plaignent d’un manque de respect et de considération à leur égard, comme en témoigne Karim :

« *À vrai dire, ma femme et mes enfants ne me respectent pas; la famille continue de vivre* [grâce à ma pension]*, mais je ne reçois aucune considération de leur part* » (Karim, 84 ans, 22 ans sous ARV).

Ces situations traduisent une fragilisation des rapports intra-familiaux souvent liée à l’histoire de la maladie et aux conflits familiaux qu’elle a pu engendrer.

Les femmes âgées vivant avec le VIH sont souvent veuves et perçoivent rarement une pension de réversion; elles dépendent financièrement de leurs enfants et vivent généralement avec l’une de leurs filles qui subvient à leurs besoins quotidiens, avec l’appui ponctuel ou régulier des autres membres de la fratrie.

Avec l’avancée en âge, les besoins des PAVVIH tendent à s’accroître, notamment en cas de maladie ou de perte d’autonomie. L’aide aux aînés est clairement déterminée par la nature et la qualité des relations entretenues tout au long de leur vie, comme une forme d’héritage des liens familiaux. Les enfants et la fratrie utérine constituent les soutiens les plus fréquemment sollicités, suivis des descendants indirects tels que les neveux et les nièces. Plus rarement, des amis de longue date ou des parents éloignés plus aisés peuvent être appelés à contribuer.

Face à la dépendance accrue liée à l’âge, les PAVVIH développent des stratégies pour limiter la fréquence de la demande et préserver la *sutura* (discrétion, dignité) – une valeur cardinale dans la société wolof – afin d’éviter la honte associée à une sollicitation jugée excessive. C’est le cas d’Adama, ancien chauffeur et chef de famille lorsqu’il était encore actif. À 73 ans et sans revenus, il vit désormais dans une situation de dépendance vis-à-vis de son fils ainé, maçon :

« *Mon fils gère la vie de la maison; s’il me donne* [quelque chose]*, je le prends; mais ma dignité ne me permet pas de lui demander quoi que ce soit* ». (Adama, 73 ans, 17 ans sous ARV).

Si les familles parviennent généralement à couvrir les dépenses de santé courantes, les coûts les plus élevés, notamment ceux liés à une hospitalisation, nécessitent souvent une plus large mobilisation, y compris des membres de la famille installés à l’étranger. Lorsque la situation s’aggrave, l’épuisement des ressources contraint parfois les familles à reporter ou renoncer à l’hospitalisation.

La situation des personnes âgées en rupture familiale apparaît particulièrement précaire. Le cas des HSH âgés en constitue une illustration. Au Sénégal comme dans de nombreux pays d’Afrique, l’homosexualité reste pénalisée et fortement stigmatisée socialement. Sur le plan épidémiologique, ce groupe est aussi l’un des plus touchés par le VIH. Nombre de HSH ont été rejetés par leur famille en raison de leur orientation sexuelle et parfois de leur séropositivité. Lorsqu’ils vieillissent et que leurs ressources diminuent, souvent sans enfants directs, ils recherchent le soutien des frères et sœurs utérins voire de leurs neveux et nièces. Mais cette mobilisation s’avère incertaine et complexe, comme l’a confié Saliou.

Saliou est âgé de 71 ans. Très jeune, il a subi violences et rejet de la part de sa famille en raison de son orientation sexuelle, ce qui l’a conduit à quitter le pays. Il a été diagnostiqué séropositif au VIH en 2001. En 2020, il a décidé de rentrer au Sénégal, mais aucun membre de sa famille n’a accepté de l’héberger. Depuis, il vit dans une grande précarité :

« *Je n’ai même pas de place pour mettre un matelas et ma valise; un ami m’héberge dans une chambre, mais quand il pleut, l’eau me tombe dessus; je souffre d’une maladie cardiaque et de diabète, j’oublie même de prendre mes médicaments; dans ma famille, personne ne veut me voir, même si je leur ai envoyé de l’argent quand j’étais à l’étranger* ». (Saliou, 71 ans, 21 ans sous ARV).

### Vieillir dans la dignité

Toutes les PAVVIH ne vivent pas dans des situations difficiles. Certaines connaissent une vieillesse paisible, entourées de leur famille, et conservent une dignité sociale. Ces cas restent toutefois rares parmi les PAVVIH rencontrées, et l’accès à un « vieillissement digne » apparaît très inégal.

Notre étude a identifié plusieurs conditions qui permettent aux PVVIH d’assumer dignement les rôles sociaux liés à la vieillesse. Tout d’abord, avoir des enfants socialement intégrés grâce à un emploi et des revenus stables est un facteur de sécurité et d’accès aux soins. Ces enfants sont en mesure de partager la charge des soins et soutenir leurs parents, qui peuvent ainsi continuer à assumer un rôle actif et reconnu au sein de la famille, comme en témoigne Ndeye.

Ndeye, âgée de 74 ans, veuve, vit avec ses deux fils, qui ont des revenus stables, ses belles-filles et ses 5 petits-enfants scolarisés. Elle dit vivre une vieillesse heureuse. De temps en temps, elle continue de pratiquer des massages à ses voisines, activité qui lui rapporte de petites sommes (500 FCFA soit 0,8 €). Ses enfants prennent soin d’elle, tandis qu’elle veille sur ses petits-enfants :

« *Je ne fais rien d’autre que m’occuper de mes petits-enfants, qui me tiennent compagnie, je suis la “yaay” (mère de famille*) ». Ndeye (74 ans, 16 ans de TARV).

D’autre part, la possibilité de parler de sa maladie à un proche, un fils ou une fille, facilite la vie quotidienne des PAVVIH. C’est le cas de Ndeye qui a informé son fils aîné mais n’a rien dit à ses autres enfants. Elle cache ses ARV dans le placard de sa chambre fermé à clé et explique ses déplacements à l’hôpital en prétextant un rendez-vous en ville. Se confier à son fils lui a permis de se libérer du poids du secret et de bénéficier d’un soutien discret. Ces confidents jouent un rôle essentiel : ils aident leurs parents à gérer leur maladie, tout en préservant le secret vis-à-vis de la famille élargie et de l’entourage social.

C’est également le cas de Mariame, qui parvient à vieillir dignement, en dissimulant sa séropositivité, grâce à l’affection de sa fille, qui est aussi sa confidente. Veuve, âgée de 72 ans, elle vit dans la maison familiale avec sa fille, son cousin et ses neveux, soit 9 personnes au total. Sa fille est la seule à connaître sa séropositivité. Elles entretiennent une relation très étroite. Cadre de profession, la jeune femme prend en charge les dépenses de santé et d’alimentation de sa mère et a recruté une jeune femme pour l’aider au quotidien.

« *Ma fille veut que je ne fasse rien. Elle me dit souvent qu’elle a engagé une femme de ménage pour que je n’aie rien à faire. Elle me demande de me reposer, et de laisser la femme de ménage s’occuper de tout* ». (Mariame, 72 ans, 20 ans sous ARVs).

Malgré la détérioration de son état de santé au cours des dernières années (elle souffre d’hypertension et d’un début de maladie de Parkinson), Mariame continue à participer à la vie sociale et remplit son rôle avec dignité.

« *J’assiste aux cérémonies. Le week-end dernier, je suis allée à Diamaguène car notre fils aîné donnait sa fille en mariage. À mon arrivée, ma fille m’a dit que je n’aurais pas dû venir à cause de mes problèmes de santé. Mais c’est normal que j’y sois, car nous devons tous y assister* ».

En définitive, les PAVVIH rencontrées qui vieillissent dignement sont celles dont les enfants sont en mesure d’apporter un soutien affectif et financier; celles qui entretiennent une relation privilégiée avec un proche jouant le rôle de confident et de gardien du secret; et celles qui ont su maintenir des liens étroits avec leur famille élargie et leur communauté. Ces conditions leur permettent d’assumer pleinement les rôles sociaux traditionnellement associés à la vieillesse et de vivre au sein d’un groupe qui les accompagnera dans cette ultime étape de leur vie.

## Discussion

Cette étude anthropologique exploratoire est la première en Afrique de l’Ouest à décrire et analyser les conditions de vie des personnes âgées de 70 ans et plus vivant avec le VIH, et suivant un traitement antirétroviral depuis plusieurs décennies.

Elle présente deux principales limites :

- d’une part, les participants étaient des PAVVIH régulièrement suivies dans des structures de santé. Ceci pourrait occulter les difficultés spécifiques des personnes moins observantes ou en rupture de soins pour diverses raisons, notamment sociales ;
- d’autre part, elle a été réalisée en milieu urbain et elle devrait être complétée par des enquêtes en milieu rural, où les infrastructures de santé sont moins nombreuses, le niveau de pauvreté plus élevé, et la structure des unités domestiques différente [44].

L’analyse des récits de vie met en évidence un ensemble d’éléments qui rejoignent ou au contraire s’écartent de ceux décrits pour des populations de même âge et d’une durée équivalente de traitement dans d’autres contextes.

Sur le plan biologique, toutes les personnes rencontrées sont en suppression virale depuis plusieurs années, signe que l’infection est stabilisée. Toutefois, le vieillissement s’accompagne de multiples comorbidités (principalement l’hypertension artérielle et le diabète) comme décrit dans toutes des cohortes de PAVVIH de 65 ans et plus aux Etats-Unis [24], en France [9], au Botswana

[30] ou en Afrique du Sud [29]. La survenue des maladies chroniques qui se combinent avec des incapacités fonctionnelles (réduction de la mobilité, troubles de la vue et de l’audition) complique le suivi médical, accroît les dépenses de santé et affecte directement la vie quotidienne et l’autonomie.

En intégrant ces dimensions biologiques aux aspects sociaux, l’approche intersectionnelle permet de comprendre la manière dont différents facteurs (biologiques, psychosociaux, économiques, genrés et culturels) interagissent avec les trajectoires individuelles. Vieillir avec le VIH apparaît comme une expérience singulière, façonnée par une combinaison évolutive de déterminants : genre, âge, stigma, état de santé, situation matrimoniale, insertion sociale des descendants, politiques publiques et fonctionnement des systèmes de santé [42]. Ces facteurs conditionnent la santé physique et mentale, l’équilibre affectif, mais aussi l’accès aux soins.

En 2024, au Sénégal, la stigmatisation sociale liée au VIH demeure un déterminant central du vécu de la maladie par les PAVVIH. Elle est renforcée par des facteurs culturels et religieux, mais aussi par la faible prévalence nationale (0,3 %), qui rend les PVVIH peu visibles socialement. Bien que la tendance ne soit pas uniforme, des études en Afrique subsaharienne ont montré le lien entre la prévalence du VIH et la stigmatisation associée [54]. Dans certains contextes, une prévalence plus élevée du VIH est associée à une stigmatisation réduite, suggérant que la normalisation de la maladie pourrait atténuer les attitudes de rejet [47].

Au Sénégal, pays à faible prévalence dans la société, la peur de la stigmatisation persiste, malgré des décennies de diffusion de messages d’information sanitaire destinés à banaliser la maladie et réduire les stéréotypes péjoratifs. Les leaders associatifs eux-mêmes hésitent à témoigner publiquement dans leur pays, préférant s’exprimer à l’international.

Chez les aînés, ces craintes se trouvent amplifiées par le tabou entourant la sexualité des personnes âgées. Dans de nombreux contextes, occidentaux comme africains, prévaut la représentation sociale de personnes âgées sans sexualité, ce qui alimente une forme d’âgisme et un sentiment de honte [5]. Les femmes âgées y sont doublement confrontées, leur sexualité étant moins bien tolérée que celle des hommes. Dans des sociétés « jeunes », comme en Afrique subsaharienne où plus de la moitié de la population a moins de 19 ans [8], le VIH est encore perçu comme une « maladie des jeunes ». Les personnes âgées dépistées à un âge avancé en conçoivent un sentiment de honte et vivent dans la crainte d’une divulgation qui pourrait compromettre le statut et le respect lié à l’âge [27]. L’intersection entre genre, âge, stigmatisation et maladie produit des configurations très différenciées [4]. Les femmes, bien que défavorisées par la polygynie et les conditions socio-économiques, sont souvent perçues comme victimes de l’infection et subissent moins de stigmatisation que les hommes, tout au moins de la part de leurs enfants. Cela leur permet d’accéder au soutien de leurs descendants lorsque ceux-ci sont adultes et socialement insérés. Pour les HSH, la situation est plus complexe. Nettement plus exposés au VIH que le reste de la population [53], persécutés du fait de leur orientation sexuelle [2], ils cumulent les stigmas et vivent dans la crainte permanente de la divulgation. Leur vieillissement dépend étroitement des configurations familiales et sociales : existence ou non d’enfants ou de neveux reconnaissants, maintien ou perte du capital économique et social après des parcours d’exil, degré de visibilité de leur orientation sexuelle.

Au-delà de la stigmatisation, les principales difficultés rencontrées tiennent aujourd’hui aux maladies chroniques et aux incapacités fonctionnelles qui altèrent la qualité de vie et alourdissent les dépenses de santé. La nécessité de consulter dans d’autres structures que les sites VIH habituels engendre un stress supplémentaire, lié au coût, à l’anonymat et à la crainte d’une divulgation involontaire.

Ainsi, le vécu différentiel du vieillissement avec le VIH dépend d’un ensemble de facteurs : genre, orientation sexuelle, ressources économiques, accès à un dispositif de protection sociale, présence d’aidants. Ces conditions sont imbriquées avec l’histoire de la séropositivité, souvent marquée par des ruptures biographiques, et avec la qualité des relations sociales et familiales construites au fil du temps. Ces contraintes limitent la reconnaissance des PAVVIH dans l’espace politique et social. Invisibles, elles restent marginalisées dans les politiques de protection sociale. Le vieillissement n’est pas encore perçu comme une priorité par les associations de PVVIH, alors même que nombre de leurs leaders historiques ont dépassé la soixantaine. Les initiatives spécifiques restent rares, malgré l’expérience acquise par ces associations dans l’accompagnement social et sanitaire.

## Perspectives de santé publique

Dans une perspective de santé publique, l’analyse des conditions de vie des PAVVIH au Sénégal suggère des interventions possibles dans différents domaines complémentaires [14,15].

Tout d’abord, il est nécessaire que les autorités de santé reconnaissent la spécificité des besoins des PAVVIH et mettent en œuvre des programmes dédiés. Un pas a été franchi dans ce sens par les autorités sanitaires du Sénégal qui, pour la première fois en 2023, ont consacré un chapitre à la prise en charge des PAVVIH dans le Plan national stratégique 2023-2030 [43].

Ensuite, l’intégration du dépistage et de la prise en charge des comorbidités et des fragilités liées à l’âge dans les services VIH doit être renforcée. Cette intégration nécessite de développer les compétences en gériatrie, de rationaliser les prescriptions et promouvoir l’usage des médicaments et réactifs biologiques à moindre coût. C’est précisément ce que teste actuellement le projet « VIHeillir : Bien vieillir avec le VIH au Cameroun et au Sénégal » [41].

Par ailleurs, le renforcement de dispositifs de couverture maladie adaptés existants (tels que le plan Sésame et les mutuelles de santé) apparaît indispensable pour réduire la vulnérabilité économique des PAVVIH. Dans la même logique, développer des mesures de soutien social sous la forme d’un revenu minimum vieillesse, renforcerait l’autonomie économique des PAVVIH, et dans la perspective d’une généralisation de cette mesure, celle des personnes âgées dans leur ensemble.

Enfin, il apparaît essentiel de renforcer les capacités des acteurs communautaires. Au Sénégal, comme dans de nombreux pays d’Afrique, les acteurs communautaires liés au VIH disposent d’une longue expérience de soutien social aux PVVIH, qu’ils ont su mobiliser de manière remarquable lors de la réponse collective à la pandémie de Covid-19. Cette expertise pourrait s’appuyer sur des initiatives comme celles du projet VIHeillir pour mobiliser diverses associations de patients et organisations de personnes âgées afin de définir de nouveaux modes d’accompagnement communautaire visant à renforcer l’inclusion sociale des PAVVIH.

Ces différentes interventions sont complémentaires, elles devraient être déployées simultanément et constituer le socle d’une politique sanitaire et sociale renforcée, en faveur de l’inclusion et de l’équité sociale pour les PVVIH.

## Conclusion

L’analyse de l’expérience contemporaine du vieillissement avec le VIH au Sénégal, à partir du point de vue de personnes âgées, révèle plusieurs éléments majeurs, aux implications à la fois théoriques et pratiques.

Comme toutes les personnes âgées du Sénégal, les PAVVIH subissent l’impact biologique et social du vieillissement. La survenue des comorbidités et des incapacités fonctionnelles réduit leur autonomie et accroît leur dépendance financière à l’égard de leurs proches. La faiblesse des dispositifs institutionnels de protection sociale et l’absence de mécanismes communautaires de soutien reportent, à l’heure actuelle, l’essentiel de la charge des soins sur les familles.

Le VIH ajoute un niveau supplémentaire de vulnérabilité. La persistance de la stigmatisation sociale du VIH continue de marquer profondément les trajectoires individuelles. Elle conduit les PAVVIH à l’auto-stigmatisation, à la crainte constance du dévoilement inopiné de leur maladie et, pour certaines personnes, à diverses formes d’autoexclusion sociale. Le maintien du secret a conditionné une part importante de leurs parcours de vie et de soins. Avec l’âge, la perte d’autonomie et les difficultés économiques obligent à solliciter l’aide de l’entourage, au risque de fragiliser des équilibres psychosociaux et relationnels longtemps stabilisés.

Dans les prochaines années, la question du vieillissement des populations en Afrique, dont on perçoit déjà les implications de santé publique, imposera de renforcer les politiques sanitaires et sociales (prise en charge des maladies chroniques, dispositifs de protection sociale) à l’échelle de chaque pays. Au cœur de ces politiques, les populations comme les PAVVIH qui cumulent les vulnérabilités, devraient faire l’objet d’un traitement spécifique. Le monde communautaire qui a montré son efficacité depuis le début de la lutte contre le sida, aura un rôle déterminant à jouer, à la fois comme lanceur d’alerte, force de propositions et de mobilisation en faveur de l’inclusion des PAVVIH.

## Déclaration éthique

Avis éthique et scientifique délivré par le Comité national d’éthique pour la recherche en santé du Sénégal, n°315/MSAS/CNERS/SP, Protocole SN21/96, version 2.0 du 07/02/2022, transmis le 16/10/2023.

## Disponibilité des données

Données ouvertes sur Dataverse : https://dataverse. ird.fr/dataset.xhtml?persistentId=doi: 10.23708/ B09IQS

## Financement

Sidaction-Ensemble contre le sida, Paris, France; 2021

## Contributions des auteurs et autrices

BT, GL-B, KS ont participé à la conception de la recherche.

L’enquête de terrain a été réalisée par SD, CF, MNN, PTS, KS, GLB et BT, supervisée par BT, GL-B, et KS.

Les analyses ont été réalisées par BT, GL-B, KS, SD, CF, MNN, PTS.

BT et GL-B ont rédigé la première version du document, révisé de manière critique et avec l’ajout d’informations substantielles par KS.

Tous les auteurs et toutes les autrices ont approuvé la version finale.

## Déclaration de liens d’intérêt

Aucun lien d’intérêt n’a été déclaré.

## Annexe : Guide d’entretien Grand âge et VIH

Guide d’entretien Grand Age et VIH

v. 17/08/2022

### 1. Eléments sociodémographiques

- Age, situation matrimoniale, nombre et âge des enfants, des petits enfants, lieu de vie, profession actuelle et passée, revenus et quelles charges assurez-vous dans la maison ?

— les relations inter et intra-générationnelles

Questions : avec qui vivez-vous à la maison ? (faire décrire précisément le nombre de personnes, le lien de parenté, l’âge approximatif, les métiers). Quelle place occupez-vous (chef de famille, membre de la famille, essayer de comprendre s’il est dépendant des personnes ou s’il occupe une place respectée). Quelles relations avez-vous avec vos frères, sœurs, époux-épouse de même génération (selon avec qui il vit). Quelles relations avec les neveux, enfants (essayer de le faire parler avec des exemples précis, des anecdotes). Quelle dépense assurez vous dans la maison ? Est-ce que la maison vous appartient ?

### 2. Biographie (à reconstituer à la fin de l’entretien, on peut aussi démarrer en demandant les grandes lignes et en y revenant par la suite)

Questions : où êtes-vous né, avec qui avez-vous grandi (parents, grands-parents..) avez-vous été à l’école, laquelle, jusqu’à quel âge ? quelle était votre profession, combien de temps l’avez-vous exercée, où, mariage (s), enfants, voyages, déménagements, grands événements de votre vie (heureux ou malheureux).

### 3. Histoire avec le VIH

Questions : quand avez-vous été dépisté, comment ça s’est passé, mise sous traitement, partage dans la famille (bref historique de la maladie et des répercussions familiales).

Actuellement, qui est informé là où vous vivez ? Comment faites-vous pour cacher les médicaments, la maladie (si pas informé).

Y a-t-il des comportements dans la famille liée au fait que vous avez le VIH (si membres de la famille informés), comment ça se manifeste ?

Est-ce plus difficile qu’avant de prendre les médicaments tous les jours ? Avez-vous des maladies que vous pensez liées aux médicaments, au VIH ?

### 4. Vieillissement avec des maladies non transmissibles

— la gestion de la multimorbidité et de la polypharmacie, intégration des nouveaux diagnostics dans la trajectoire de vie

Questions : De quelles maladies souffrez-vous en dehors du VIH ? HTA, diabète, hépatite, maladie cardiaque, autre ? (faire raconter l’itinéraire thérapeutique de chaque maladie : quand elle a été diagnostiquée, où, par qui, examens, traitements, coûts des traitements, hospitalisation ?) est-ce que ça a été difficile pour vous d’apprendre que vous aviez (HTA, diabète…) ? qu’avez-vous ressenti quand le médecin vous l’a dit ? quelle est la maladie actuellement qui vous cause le plus de souci ?

Quels sont les médicaments que vous prenez (pour chaque maladie décrire le médicament, le nombre de comprimés, de prises, les difficultés rencontrées) ?

Est-il plus facile de prendre les ARV que les autres médicaments, ou préférez-vous ? pourquoi ?

### 5. Mobilisation des ressources

— Quels sont vos revenus (pensions, aides familiales, métier) ? Estimez-vous que c’est suffisant pour vos besoins alimentaires, de soins ?

— la mobilisation des solidarités traditionnelles

Questions : Arrivez-vous à acheter les médicaments dont vous avez besoin ? Quand vous avez besoin d’argent, pour vous soigner, à qui faites-vous appel en premier ? Est-ce différent selon le type de dépense ? (faire décrire à qui il s’adresse en premier, dans quel cas, en second, dans quel cas etc.). La dernière fois où vous avez eu un problème, à qui avez-vous demandé ? pourquoi ? est-ce que la personne vous a aidé ? Différences entre hommes et femmes ?

— l’utilisation des dispositifs modernes de protection sociale

Questions : Quand vous avez été malade la dernière fois, (quelle maladie) avez-vous utilisé le plan Sésame ? Si oui quelle a été la procédure ? Sinon pourquoi ? Etes-vous à l’IPRESS, avez-vous une mutuelle ? (faire raconter une expérience réussie ou non de prise en charge)

### 6. L’expérience et les perceptions des situations de dépendance physique et psychique

**—** vécu des situations de dépendance par les personnes âgées, les familles et stratégies pour les éviter ou les retarder : faire raconter par les personnes la situation de dépendance physique ou psychique, les difficultés et souffrances que cela engendre et ce que les accompagnants ont mis en place pour faciliter la vie.

Pour les personnes dépendantes : avez-vous besoin d’aide au quotidien, pour quoi faire ? a qui demandez-vous de l’aide, pour quel type d’aide (toilette, se déplacer, manger, payer des soins). Est-ce difficile ou est-ce naturel dans les relations avec les membres de la famille ? (faire raconter le type d’aide et la relation que cela induit).

— redéfinition des relations inter-générationnelles dans un contexte de dépendance

Questions aux personnes en situation de dépendance : qu’est-ce qui a changé pour vous depuis que vous avez besoin d’aide pour votre vie quotidienne ? quelle est l’attitude de vos enfants, petits-enfants, neveux à votre égard ?

Pour les accompagnants : quelle aide fournissez-vous ? est-ce difficile pour vous ? pour lui ? qu’est-ce que ça a changé dans votre relation ? y arrivez-vous, avez-vous le sentiment d’être dépassé (questions à poser uniquement si la personne semble avoir des difficultés). Comment vous êtes-vous organisé pour prendre en charge la personne (répartition des charges dans la famille, faire raconter qui paie, qui l’accompagne à l’hôpital, qui passe la nuit avec, qui l’aide au quotidien etc.) Avez-vous vécu des situations difficiles, lesquelles (par exemple hospi et impossibilité de payer), ou dépendance physique et personne à la maison pour l’assister)

— Mécanismes de résilience mis en œuvre pour réorganiser le quotidien

Comment faites-vous au quotidien pour vous faire aider, avez-vous pu gérer le changement ? Comment ?

Accompagnants : comment vous êtes-vous organisé dans la famille pour gérer la dépendance ?

— stratégies de structures de santé ou éviter, retarder et gérer les situations de dépendance des personnes âgées

Questions aux soignants : dans vos patients, avez-vous des personnes en situations de dépendance physique ou psychique (faire raconter des situations précises). Comment les avez-vous géré avec la famille ? Que faites-vous pour retarder, éviter, gérer ces situations (selon le professionnel, lui faire raconter ce qu’il a fait – prescription pour les médecins, mise en place d’aide pour les assistants sociaux, médiations familiales- ce qui a marché, ce qui a moins bien marché)

— Existence/utilisation d’appui aux personnes et aux familles

Avez-vous des aides en dehors de la famille, si oui qui ? au niveau des institutions ? Lesquelles ? Comment ça se passe ?

### 7. Expériences et perception du vieillissement <aborder plus tardivement dans l’entretien>

— Les expériences du vieillissement, la transition vers le statut de « personne âgée »

Questions : Avez-vous le sentiment d’être vieux ? Vous définissez-vous comme « vieux », « personne âgée », autre ? (quel mot en wolof) ? A quel moment vous êtes-vous senti vieillir? Pensez-vous à la mort, sentiment de peur ? (voir comment aborder la question, par une relance si c’est lui qui l’évoque, ne pas poser la question directement)

Vie quotidienne : quelles sont vos activités quotidiennes, comment passez-vous vos journées, y a-t-il des choses que vous voudriez faire et que vous ne pouvez-vous plus faire ?

— les éléments constitutifs d’un « vieillissement réussi »

Questions : pour vous, c’est quoi un « vieillissement réussi » ? (lui faire dire les éléments, relancer pour avoir plusieurs éléments constitutifs)

— Les changements individuels, familiaux et sociaux

Questions : Qu’est-ce que le fait de vieillir a changé dans votre vie, dans les relations avec votre famille, vos amis, votre vie sociale, professionnelle, la vie sexuelle (voir comment aborder la sexualité, savoir s’il ou elle est sexuellement actif, si cela l’épanouit ou si l’absence de sexualité lui manque) ? Les personnes de votre famille et de l’entourage ont-ils changé de comportement depuis que vous avez pris de l’âge ? Ressentez-vous une mise à l’écart depuis que vous êtes « vieux » ? Comment ça se manifeste, quand ça a commencé, qui sont les personnes qui le mettent à l’écart ? Est-ce lié à une perte de revenus ? (stigmatisation liée à l’âge)

– Les différences entre les hommes et les femmes âgées : Quelle différence ? dans les activités quotdiennes, dans les activités sociales, dans le rapport avec les institutions médicales ?

### 8. Les modalités d’adaptation des structures de santé et des structures communautaires (associatives) aux populations âgées vivant avec le VIH et co-morbidités

– Perception de l’efficacité du dispositif de prise en charge sur la plan sanitaire et social des Personnes âgées vivant avec le VIH

Questions : Faites vous partie d’une association de PVVIH ? Cela vous a-t-il aidé ? En quoi (faire raconter ce que l’asso a fait pour lui).

Comment se passe votre suivi dans le site de prise en charge ? Vous sentez-vous appuyé en tant que personne âgée ? Quels sont les aides qui vous ont été proposées, pour quelle maladie ou situation ?

Soignants : Mettez-vous en place des services particuliers pour des PVVIH âgés ? lesquels ? vous semblent-ils efficaces ? Quels sont les problèmes particuliers que rencontrent les PVVIH âgés et quelles sont les interventions que vous avez mises en place ?

— Intégration du VIH dans les services gériatriques et de la gériatrie dans les services de prise en charge du VIH

Soignants service gériatrie : avez-vous des personnes VIH+ ? Ont-elles des difficultés particulières ? comment les gerez-vous (essayer de faire raconter une expérience d’un PVVIH hospitalisé ou suivi). Cela vous semble difficile, en quoi, quelles adaptations avez-vous mis en place ?

Soignants services VIH : Quelles difficultés rencontrez-vous avec les PVVIH âgées ? Avez-vous mis en place des actions particulières ? (assistance sociale, aide au plan Sésame, médiation familiale, adaptation médicaments….)

PVVIH âgés : où vous faites-vous soigner pour vos maladies (faire décrire pour VIH, diabète, HTA….). Quelles difficultés à vous faire suivre à différents endroits (divulgation statut VIH ou non, coût des déplacements, médecins différents….)

— Acceptabilité et efficacité du modèle VIHeillir

Pour les patients de VIHeillir : êtes vous dans le projet VIHeillir ? qu’est ce que ça vous apporte ? Quand venez vous en consultations (tous les 3 mois, 6 mois), participez-vous aux activités sociales (lesquelles, faire raconter son expérience). Sinon est-ce parce qu’on ne vous l’a pas proposé ? Vous n’avez pas voulu, pourquoi (indisponibilité, coût du transport, peur de la divulgation….)

Quel traitement prenez vous en dehors du VIH, combien de comprimés par jour au total, estce difficile, en quoi ? Combien payez-vous par mois pour le traitement ?

— Rôle des associations de PVVIH dans l’accompagnement des PAVVIH

Faites vous partie d’une association de PVVIH, laquelle, que vous apporte-t-elle ? d’une autre asso (diabète, HTA), que vous apporte-t-elle ? Sinon, pourquoi, manque d’intérêt, pas de proposition/occasion ?

### 9. Les répercussions de l’épidémie de Covid-19 sur les personnes âgées vivant avec le VIH

– Vécu de l’épidémie et des mesures sanitaires liées à l’épidémie Covid par les PVVIH âgées

Avez-vous ressenti de la peur lors de l’épidémie Covid, pourquoi, vous êtes vous senti vulnérable ? par rapport au VIH, aux autres maladies ? Les mesures de l’état (état d’urgence, restrictions) vous ont-elles paru normales, anormales, était-ce difficile ? Quel vécu dans la famille (faire raconter comment ça s’est passé pendant le Covid, sur la vie quotidienne, les soins VIH, les autres soins ou maladies, répercussion économiques…)

— Impact sur les relations familiales

Le Covid a-t-il changé quelque chose dans les relations avec la famille, quoi ? (par exemple y avait-il des polémiques avec les jeunes sur le respect des mesures, ou au contraire une attention de la famille vis-à-vis des vieux ?)

— Répercussions sur l’accès aux soins et aux traitements

Comment avez-vous fait pour récupérer les ARV, asso dispensation à domicile, venus en consultations, quelles difficultés, idem pour diabète et HTA

— Dispositifs mis en œuvre par les structures de santé et les associations de PVVIH pour pallier les difficultés

Y a-t-il eu une organisation particulière au moment du Covid dans votre site de suivi VIH et pour les autres maladies ? Laquelle, comment l’avez-vous vécu ? (report de RDV, difficulté d’avoir une consultation ou au contraire dispensation à domicile…)

### 10. L’impact du vieillissement, des co-morbidités et des situations de dépendance spécifiquement et comparativement, chez les femmes et les hommes âgés vivant avec le VIH -> cet aspect est issu de l’analyse de l’entretien

— Spécificités de genre liées au vieillissement avec le VIH

— Influence des rôles définis par la société sur le vécu du vieillissement avec le VIH

— Conditions de la qualité du vieillissement en fonction du genre

— Vécu différentiel du vieillissement par les hommes et les femmes en situation de couple
