# Supplementary material for: Growing old with HIV in Senegal: an anthropological perspective for a public health policy
Source: Med Trop Sante Int. 2026 Feb 27;6(1):mtsi.v6i1.2026.769. [Article in French] doi: 10.48327/mtsi.v6i1.2026.769 (PMC13216905; doi:10.48327/mtsi.v6i1.2026.769)
Supplement: Related texts

# Research article: Growing old with HIV in Senegal: an anthropological perspective for a public health policy

## Introduction

### International context

The biological efficacy of antiretroviral therapy (ART) has greatly impacted the lives of people living with HIV, transforming the prognosis of the disease from fatal to chronic. In just a few years, life expectancy for people living with HIV (PLHIV) has become similar to that of the general population [32,50]. This development has led to the gradual aging of patient cohorts, including in Africa [1,34]. As early as 2013, UNAIDS reported an increase in the number of people aged 50 and older living with HIV (OPLHIV). This increase was attributed to the success of ART, the decline in incidence among young people, and the persistence of risky behaviors among older people [39]. In 2022, in line with these projections, OPLHIV accounted for over 50% of PLHIV in high-income countries and 30-35% in sub-Saharan Africa [15]. The number of OPLHIV in these regions was estimated at 4 million in 2011 and is expected to reach approximately 9 million by 2040 [26].

UNAIDS and various authors have recommended new policies for prevention, testing, and treatment, including those for noncommunicable diseases, that are tailored to OPLHIV [1,39]. However, countries in West and Central Africa have been slow to implement these policies [22]. Furthermore, the aging of PLHIV is part of a broader demographic trend. In sub-Saharan Africa, for example, the population aged 65 and over is expected to triple from 34 million to 98 million between 2022 and 2050 [33]. This trend will put additional strain on health systems, which are currently ill-prepared [48]. In the absence of robust policies for older adults [28], support for the elderly relies mainly on families, many of whom live in chronic poverty [20]. Therefore, the living conditions of older people remain precarious, and their access to care is often limited. According to the WHO, aging with dignity requires autonomy, respect, a decent quality of life, access to appropriate care, and emotional support and social recognition [40]. This ideal is still far from being achieved in sub-Saharan Africa.

### Context in Senegal

In Senegal, the first French-speaking African country to implement a government program providing access to ART in 1998 [10], context is important. Since 2003, ART and viral load testing have been free of charge, but patients are responsible for some of the care costs. The HIV epidemic in Senegal is concentrated, with low prevalence in the general population (0.3%) and high prevalence in certain areas and among vulnerable populations. In 2022, 34% of the 32,000 people receiving ART were 50 years of age or older [43].

Socioeconomically, approximately 38% of Senegal’s population and half of households headed by older individuals live below the poverty line [44]. Social policies are limited; only 20% of older adults receive retirement pensions. The Plan Sésame, the main institutional support for older people created in 2006, aims to provide free care to those over 60 without other medical coverage. However, its implementation remains uneven due to delays in reimbursement by the state to health facilities. In practice, older people bear a large share of health care expenses [49]. Family support remains their primary source of assistance, especially since it is rooted in the social ethic of intergenerational solidarity [12]. Intergenerational cohabitation is common in Senegal [44], promoting inclusion but testing the limits of solidarity due to the economic instability of households. In a context of polygamy, gender inequalities are pronounced: nearly half of women over 70 are widows, compared to 3% of men. Their situation is often precarious [19]. In a predominantly Muslim society (95%), social norms value old age and encourage religious sentiment among the elderly [3,18]. Despite this social recognition, their living conditions remain difficult [23,36,37]. HIV remains a stigmatizing disease, especially since its low prevalence makes PLHIV less visible [45]. In this context, aging with HIV is a significant challenge.

### Anthropological research in Senegal

Since the 2000s, a wealth of anthropological literature has documented the social, cultural, and political conditions affecting access to ART in Africa [13,35,46] and their impact on PLHIV [11,54]. Additionally, there is a substantial body of literature on aging and caregiving relationships in Africa [17,18,28,31]. Nevertheless, anthropological studies on aging with HIV in French-speaking Africa are still rare. Questions remain: How do aging and HIV infection interact in the context of African societies? What are the experiences and perceptions of OPLHIV and their families when faced with aging and situations of dependency? What health and social responses are provided by relatives and institutions?

As part of the Old Age and HIV project, funded by Sidaction, a study was conducted in Dakar to analyze the living conditions and care relationships of OPLHIV.

## Methods

### Study dates and sites

The study took place between June 2022 and April 2023 in three health facilities: the Outpatient Treatment Center (OTC) and the Fann Clinical Care, Research, and Training Center (FCCR), both of which are located in the Fann National University Hospital Center in Dakar; and the Baye Talla Diop Health Center, also known as “Dominique,” in the suburbs of Dakar.

Established in 1995 and 2005, respectively, the OTC and FCCR are national and international reference centers dedicated to the medical and social care of PLHIV. They are involved in HIV training for most of the country’s healthcare workers. Approximately 3,000 PLHIV are monitored there, nearly 50% of whom are over 50 years of age. The third site is a multipurpose urban medical facility where approximately 450 PLHIV are monitored. At each of these sites, mediators – who are members of PLHIV support associations – participate in the psychosocial care of PLHIV.

### Study population

The study involved people living with HIV who were 70 years of age or older. At this age, the onset of comorbidities and functional disabilities can lead to reduced autonomy and dependence [16]. Participants were selected through a reasoned process to ensure diversity in terms of gender, marital status, sexual orientation, economic situation, and care site.

The number of interviews was determined based on the principle of information saturation [25]. A total of 31 OPLHIV were included in the study (14 women and 17 men), with a median age of 73 (range 70–90). Half of the participants had been diagnosed with HIV before 2005, with the earliest diagnosis dating back to 1987. The median duration of antiretroviral therapy was 16 years (range, 6–22 years), and eight participants had received treatment for more than 20 years.

Interviews were also conducted with five doctors (including a geriatrician), a nurse, four community mediators, and six family caregivers. Additionally, informal interviews were conducted with national leaders in the fight against AIDS and various community members, including associations of PLHIV, associations involved in caring for other diseases (e.g., stroke, diabetes, and hepatitis), and the country’s main organization for older people: the National Council of Seniors of Senegal. A total of 51 people were interviewed.

### Data collection method

Information was collected through semi-structured interviews and direct observations. The study was conducted by: (i) two Senegalese research assistants, one with a master’s degree in community health and the other with a master’s degree in sociology; (ii) a community mediator who is a member of an association of women living with HIV and has worked with PLHIV in Dakar for nearly 25 years; and (iii) three senior anthropologists, two of whom are also doctors, with 15 to 30 years of experience researching HIV in West Africa.

Of the 26 interviews with OPLHIV, most took place at care facilities at the respondents’ request, mainly for reasons of confidentiality. However, five interviews took place at home, which allowed us to observe the respondents’ living conditions. Participants were given a transportation allowance when the interview took place at a health facility, and small gifts, especially food, were offered during home visits. In situations of extreme poverty, one-time support was provided to purchase medication, for example.

The interviews used a thematic guide (see Appendix) for each category of interviewee and explored the following topics: life history, medical history, treatment history, experience with the disease, experience of family members, intra-family relationships, support networks, and organization of care. These themes were developed and refined over time based on topics raised by interviewees or questions raised during narrative analysis sessions.

Following the patient interviews, with consent, information obtained was supplemented by information from social and medical professionals, particularly bioclinical information extracted from medical records. Interviews with healthcare professionals were conducted in French. All others were conducted in the local language, Wolof, and translated and transcribed into French. Ethical approval for the study was obtained from the Senegalese National Ethics Committee for Health Research. Each participant was given a detailed explanation of the study protocol and had the opportunity to ask questions before giving their verbal consent. Participation in the interviews was entirely voluntary. All team members were trained in research ethics and signed confidentiality agreements. During transcription, each participant was assigned a pseudonym to ensure anonymity. All first names mentioned in the quotations and life story excerpts in this article are fictitious.

### Theoretical framework

Our theoretical approach is based on three key concepts. The first is the concept of “transition” as defined by Grenier [21], from a life course perspective. This concept highlights the fluctuating, evolving, and unique nature of the “transitions” that mark our existence. The second concept is the intersectional perspective, inspired by Crenshaw [6], which describes the intertwining and interdependence of the multiple, unequal social relationships in which individuals are involved. The third concept is that of “the ethics of care” , which Tronto defines as a set of moral values and practices aimed at supporting people who cannot live independently [51]. This concept distinguishes four stages in this process: recognizing the needs of others (care about), assuming responsibility for responding to those needs (taking care of), actually providing care (care giving), and receiving care (care receiving).

This approach allows us to analyze when and how care is provided and its contribution to the well-being of the individual [52]. It also sheds light on the care practices implemented by family, community, and institutional actors in supporting OPLHIV.

The three concepts–life course transitions, intersectionality, and the ethics of care–are closely interlinked. This interconnection is essential to understanding how biological and social determinants influence the experience of aging with HIV in different ways.

### Method of analysis

The data corpus (interviews and observations) underwent inductive content analysis inspired by grounded theory. This qualitative approach aims to derive categories of analysis directly from empirical data. Manual data coding was carried out iteratively as the study progressed, allowing new themes to be identified in addition to those already identified [38].

Inspired by Grenier’s “transition in life course” [21], we developed a biographical approach designed to reconstruct each individual’s life narrative, situating them in their historical journey from birth to the present in the family, professional, and medical spheres. This approach allows us to analyze how each person interprets their existence and their relationships with family members and others. Thus, it offers the possibility of grasping the social in its individual form–key life events shaped by collective dynamics (education, work, parenthood, etc.)–while placing them in an overall social context marked by broader cultural, economic, and political conditions (e.g., the political and historical framework, collective norms, and values) [7].

Senior anthropologists and research assistants conducted the analyses from an educational perspective. The preliminary results were then discussed with members of community associations of PLHIV to strengthen their participation in the research process.

## Results

### HIV and ART, a peaceful experience

All the OPLHIV interviewed described a form of habituation to their disease:

“*We live with it. It’s no longer a problem. We get used to it. We almost forget that we are sick*” (Aminata, 70 years old, 21 years on ART).

Most of these individuals were diagnosed at an advanced stage of the disease, already suffering from various opportunistic infections. Others were diagnosed with HIV only after experiencing symptoms they considered minor, such as fatigue, cough, or fever. In all cases, the diagnosis was experienced as a tragedy. Many people remember sharing with their loved ones the belief that they would die quickly. They all mention the distress caused by stigma because HIV was perceived as a disease affecting gay men and sex workers. Amadou is 70 years old. A former fishing boat mechanic, he traveled along the coast of West Africa. At age 55, he was hospitalized for a lung abscess and discovered he was HIV positive.

“*At the time, I thought my life was over*,” he says. After 15 years of ART, he states the following in 2022, at the time of the interview: “*To me, HIV is not a disease. A disease keeps you bedridden, but HIV doesn’t prevent me from doing what I want. It doesn’t tire me. I just take my medication* ” (Amadou, 70 years old, 15 years on ART).

Like Amadou, all of the interviewees expressed feeling as though they had overcome the ordeal. Thanks to ART, their health improved dramatically. The disappearance of the most visible stigmas associated with the disease, particularly extreme thinness and diarrhea, has enabled them to return to a sense of normalcy, both physically and socially. All respondents testified to the help they received from community mediators and associations.

The difficulties associated with early treatments are now a thing of the past. In the early 2000s, antiretroviral treatment regimens involved taking up to 20 pills a day and caused debilitating side effects, including nausea, diarrhea, and insomnia. Thanks to medical advances, these treatments are now usually limited to a single pill per day with fewer side effects.

All of the OPLHIV interviewed emphasized their exemplary compliance with their ART, and this was confirmed by healthcare professionals. They say they have returned to a “normal” life. Those who benefited from the initial treatments in the early 2000s consider themselves true survivors.

### Silence, secrecy, sharing

Although the OPLHIV in the survey say they have regained a form of social normality, their living conditions are still marked by the fear of the social stigma associated with HIV.

“*HIV is a disease that must not be disclosed. It is not a pleasant disease to have*,” (Ndeye, 74 years old, 16 years on ARVs).

The circumstances of the diagnosis often determined how it was disclosed. For instance, when a patient was hospitalized in serious condition, healthcare professionals would often disclose the diagnosis to a family member without consulting the patient. In most cases, however, patients were informed directly and encouraged to share the diagnosis with a loved one who could provide material or moral support. These confidants were usually spouses, people who accompanied patients to appointments, or people who financed their care. Women often preferred to inform a mother or sister. Widows were more inclined to share the information with their adult children because they were convinced that they had contracted HIV from their deceased husbands and were anxious not to be accused of misconduct.

The fear of moral judgment about the circumstances of the infection remains the central factor guiding the choice of whom to inform and when to disclose. Men disclosed their HIV status less frequently, sometimes to their wives, sisters, or brothers. Disclosure within couples was more common at the time of diagnosis than during subsequent remarriages. Over time, disclosure became less common. Ten or twenty years after diagnosis, some PLHIV still remain completely silent about their illness.

Among men who have sex with men (MSM), this reluctance is even stronger. Paul (70 years of age) hid his homosexuality for a long time. He married to avoid suspicion; he became the father of a son; then he divorced after a family scandal caused by the discovery of his sexual orientation. Rejected by his family, Paul left Senegal but returned in 2010 when he was diagnosed with HIV. He decided to keep his illness secret, especially from his son.

“*I did everything for him, everything. But I tell myself that in his eyes, I am a disgrace*” (Paul, 70 years old, 14 years on ART).

Karim, an 84-year-old retired mechanic, embodies a different kind of silence. He lives with his wife, two children, and grandchildren. He has never openly discussed his illness with his children in order to preserve his dignity.

“*There are some conversations I will never have with my children. I have never talked to them about my illness. They know about it because my eldest daughter accompanied me to the hospital in 2000. I know they are aware of it, but I have never talked to them about it*” (Karim, 84 years old, 22 years on ART).

The taboo surrounding sexuality among older people exacerbates the reluctance of those diagnosed at an advanced age. Older women are often widowed, having lost their spouses to the disease or due to age differences in the context of polygamy. They have all been under family pressure to remarry, but none have agreed, fearing that their new spouse would reveal their illness. However, the onset of age-related disabilities (blindness and reduced mobility) introduces new dilemmas. Dependence on others for daily activities, such as taking medication or traveling to medical appointments, sometimes forces them to reevaluate their initial choices and lift the veil of secrecy. This revelation can clarify what has been left unsaid and elicit compassion. In other cases, it revives past conflicts and accusations of concealment.

### The onset of comorbidities

The people in the study now have a peaceful relationship with HIV. All of them have maintained an undetectable viral load for several years. They emphasize the importance of taking ART regularly and undergoing biological monitoring. As people age, their concerns shift. It is no longer HIV and its treatment that cause worry, but rather the onset of other chronic diseases and functional disabilities. The most common are high blood pressure, diabetes, and its complications, such as stroke, kidney failure, heart disease, and blindness, as well as osteoarticular pain. These diseases complicate medical follow-up and require new care pathways. They also necessitate consultations with specialists outside of the usual HIV care structures. One of the main sources of concern is financing additional tests and medications for these chronic conditions.

“*I suffer from high blood pressure. The doctor prescribed medication for me. I used to buy it at the end of each month. But it’s too expensive. I struggle to find the money to buy it. So, I’ve had to stop taking it because I can’t afford it.*” (Aminata, 70 years old, 22 years on ART).

The cumulative costs quickly add up to significant sums that are difficult to raise over the long term. For people with multiple conditions, taking additional medication complicates their daily lives. Out of habit and conviction, some prioritize their ART over other medications that are considered less important.

### Economic decline and financial insecurity

The life stories collected show that HIV infection has caused major upheaval in people’s lives. The disease has led to various forms of disruption, including job loss, divorce, widowhood, and family conflicts. Medical care and treatment have enabled a gradual return to relative stability, allowing people to re-invest in their social roles. However, with advancing age, this balance becomes fragile once again. Reducing or ceasing professional activities due to retirement or functional disabilities marks a new biographical rupture.

For people in the formal sector, retirement pensions remain modest. The highest amount reported in the study is 35,000 CFA francs per month (€53). Widows receive even lower amounts under survivor’s pension schemes (25,000 CFA francs/month, or €38), especially in polygamous households where the pension is divided among the wives.

Most people had worked in the informal sector. Without a pension or regular income, some sold their possessions to cover healthcare expenses, while others were forced to repeatedly move, which gradually pushed them to the urban periphery in search of more affordable rents. As Mohamed explains, most of the OPLHIV interviewed said they had sought work for as long as their physical condition allowed.

“*I worked for years in a fish factory unloading crates, but I had to stop three years ago because it was too hard*.” (Mohamed, 74 years old, 19 years on ART).

### Who cares for OPLHIV?

The study results show that PLHIV face various forms of dependency that gradually extend to all areas of daily life. This forces them to seek different types of support, mainly in three forms: healthcare facilities, PLHIV associations, and families.

### Healthcare facilities

As part of HIV infection monitoring, biannual visits to healthcare facilities punctuate the lives of the OPLHIV we met. These visits allow them to renew their ART and undergo biological testing. These appointments are integrated into their daily lives, and these facilities are perceived as places of trust and recognition. Respondents say they feel respected and welcome there. Over the years, they have formed personal bonds with healthcare professionals who call them by familiar and respectful nicknames, such as “Uncle Faye” or “Aunt Fanta.” These relationships sometimes offer privileges, such as avoiding lines, receiving certain tests free of charge, and receiving food donations. Patients are sometimes invited to participate in celebrations of national events organized at these facilities, such as World AIDS Day and Pink October. During these events, patients are honored with genuine affection, though sometimes tinged with a hint of condescension. In the eyes of healthcare workers, their presence is a testament to the quality of care and success of management.

Even when travel is long and costly, OPLHIV do not consider seeking care elsewhere. Fatou, who has received treatment for 20 years at a referral hospital in Dakar, exemplifies this commitment. Although health centers closer to her home provide ART, she insists on coming to the hospital:

“*I prefer to come here because they specialize in HIV, and I’m not worried about confidentiality here, unlike at the health center. We only talk about our illness here. It’s the only place where we talk about it and see our peers*” (Fatou, 70 years old, 20 years on ART).

Caregivers at these facilities adopt a protective attitude and often consider themselves a “second family” for their patients, whom they have treated for years.

However, when people require referral to specialized services for other chronic diseases such as hypertension or diabetes, they have a contrasting experience. They are anonymous in endless lines, faced with the high costs of consultations, additional tests, and medication. They struggle to find the same quality of relationship. The fear of stigmatization sometimes leads them to conceal their HIV infection, accentuating their feelings of vulnerability.

### PLHIV associations

Since the 2000s, PLHIV associations have played a central role in prevention, counseling, screening, and patient support. Some of their members act as mediators within healthcare facilities. During our interviews, the leaders of these associations indicated that they were aware of the growing difficulties faced by their aging members. However, no specific measures have been put in place for them. It is only since the 2020 Covid epidemic that targeted initiatives have emerged. The first was home delivery of ART to compensate for travel restrictions imposed by health measures. This service has since been maintained, and several organizations now offer it to older people who have difficulty accessing healthcare facilities to obtain their ART.

### Family support

The daily care of OPLHIV is shared among different members of the household. In line with the gendered division of household tasks, women (daughters, daughters-in-law, and nieces) most often provide care for dependent older people. Other family members living nearby or abroad may also be called upon for support, particularly financial.

The testimonies of OPLHIV in our study show that family support configurations vary according to gender and socioeconomic status. Among older married men living with HIV, wives appear to be the main providers of daily care. They are often much younger and may or may not be aware of their spouse’s illness. Some are themselves living with HIV. Regardless of their age, men typically maintain their role as head of the household as long as they receive a retirement pension or have a steady income. In a context marked by high youth unemployment, this pension becomes an essential household resource. It is often used for the benefit of the entire family, sometimes at the expense of the elderly’s needs.

Ousseynou, a former teacher who lives in the family home, is the only one with a regular income.

*“I pay the household bills. When my pension arrives, I pay the water and electricity bills, as well as all the daily expenses because the children don’t work. My pension isn’t enough, so I make do*” (Ousseynou, 81 years old, 11 years on ART, 10 dependents).

Nevertheless, some elderly men complain of a lack of respect and consideration, as Karim testifies:

“*To tell the truth, my wife and children don’t respect me. The family continues to live thanks to my pension, but I receive no consideration from them*” (Karim, 84 years old, 22 years on ART).

These situations reflect weakened intra-family relationships, which are often linked to the history of the disease and the conflicts it may have caused within the family.

Older women living with HIV are often widowed and rarely receive survivor’s pensions. They are financially dependent on their children and generally live with one of their daughters, who provides for their daily needs with occasional or regular support from other siblings.

The needs of female OPLHIV tend to increase as they age, particularly in the event of illness or loss of independence. The support available to older people is determined by the nature and quality of the relationships they have maintained throughout their lives. These relationships serve as a legacy of family ties. Children and siblings are the most frequently sought-after sources of support, followed by indirect descendants, such as nephews and nieces. Less frequently, long-time friends or wealthy distant relatives may be called upon for support.

Faced with increased dependence due to age, OPLHIV develop strategies to limit the frequency of requests and preserve *sutura* (discretion and dignity)–a cardinal value in Wolof society–to avoid the shame associated with excessive solicitation. This is the case for Adama, a former driver and the former head of his household. Now 73 years old and without an income, he depends on his eldest son, a mason. Adama says:

“*My son manages the household. If he gives me something, I take it, but my dignity does not allow me to ask him for anything*.” (Adama is 73 years old, 17 years on ART).

While families can generally cover routine healthcare expenses, higher costs, particularly those related to hospitalization, often require greater mobilization, including from family members living abroad. When the situation worsens, the depletion of resources sometimes forces families to postpone or forgo hospitalization.

The situation of elderly people who have been cut off from their families is particularly precarious. The case of elderly MSM is a case in point. In Senegal, as in many other African countries, homosexuality is still criminalized and socially stigmatized. Epidemiologically, this group is also one of the most affected by HIV. Many MSM have been rejected by their families because of their sexual orientation, and some have been rejected because they are HIV-positive. As they age and their resources dwindle – often without children of their own – they seek support from their siblings or even their nieces and nephews. However, this support is uncertain and complex, as Saliou explained.

Saliou is 71 years old. He suffered violence and rejection from his family at a very young age because of his sexual orientation, which led him to leave the country. He was diagnosed with HIV in 2001. In 2020, he decided to return to Senegal, but none of his family members agreed to take him in. Since then, he has been living in extreme poverty.

He says, “*I don’t even have a place to put a mattress or my suitcase. A friend lets me stay in a room, but when it rains, water drips on me. I have heart disease and diabetes, and sometimes I forget to take my medication. No one in my family wants to see me, even though I sent them money when I was abroad*.” (Saliou, 71 years old, 21 years on ART).

### Aging with dignity

Not all OPLHIV live in difficult situations. Some enjoy a peaceful old age surrounded by their families and retain their social dignity. However, these cases remain rare among the OPLHIV we met, and access to “dignified aging” appears to be uneven at best.

Our study identified several conditions that enable PLHIV to assume the social roles associated with old age with dignity. First, having children who are socially integrated through stable employment and income provides security and access to care. These children can share the burden of care and support their parents, enabling them to continue playing an active and recognized role within the family. Ndeye’s story exemplifies this.

Ndeye is a 74-year-old widow who lives with her two sons and their wives, as well as her five grandchildren, all of whom are in school. She says she is enjoying a happy old age. From time to time, she gives massages to her neighbors for small amounts of money (500 CFA francs, or €0.80). Her children take care of her while she looks after her grandchildren.

“*I do nothing but take care of my grandchildren, who keep me company. I am the* yaay *(mother of the family)*.” (Ndeye, 74 years old, 16 years on ART).

On the other hand, being able to talk about their illness with a loved one – a son or daughter, for example – makes daily life easier for OPLHIV. This is the case for Ndeye, who told her eldest son but not her other children. She hides her ART in her locked bedroom closet and explains her trips to the hospital by saying she has appointments in town. By confiding in her son, she has freed herself from the burden of secrecy and gained discreet support. These confidants play an essential role by helping their parents manage their illness while keeping the secret from the extended family and social circle.

Mariame is also able to age with dignity while hiding her HIV status thanks to her daughter’s affection and support. Mariame is a 72-year-old widow who lives in the family home with her daughter, cousin, and nephews–a total of nine people. Her daughter is the only one who knows about her HIV status. They have a very close relationship. The young woman, a professional executive, takes care of her mother’s health and food expenses and has hired someone to help with daily tasks.

“*My daughter wants me to do nothing. She often tells me that she hired a housekeeper so that I wouldn’t have to do anything. She asks me to rest and let the housekeeper take care of everything*.” (Mariame, 72 years old, 20 years on ART).

Despite her health deteriorating in recent years – she suffers from high blood pressure and early-stage Parkinson’s disease – Mariame continues to participate in social life with dignity.

“*I attend ceremonies. Last weekend, I went to Diamaguène because our eldest son was marrying off his daughter. When I arrived, my daughter told me that I shouldn’t have come because of my health problems. But it’s normal for me to be there because we all have to attend*.”

Ultimately, the OPLHIV who are aging with dignity are those whose children can provide emotional and financial support, those who have a close relationship with a confidant and keeper of secrets, and those who have maintained close ties with their extended family and community. These conditions enable them to embrace the social roles traditionally associated with old age and live among a group that will support them in this final stage of life.

## Discussion

This exploratory anthropological study is the first in West Africa to describe and analyze the living conditions of people aged 70 and over who are living with HIV and have been undergoing antiretroviral treatment for several decades.

It has two main limitations:

- First, the participants were OPLHIV who were regularly monitored in health facilities. This could obscure the specific difficulties of people who are less compliant or have discontinued treatment for various reasons, particularly social ones.
- Second, it was conducted in an urban setting and should be supplemented by surveys in rural areas, where health infrastructure is less available, poverty levels are higher, and household structures differ [44].

Analysis of the life stories highlights elements that either correspond to or differ from those described for populations of the same age with an equivalent treatment duration in other contexts.

Biologically, all of the individuals interviewed have experienced viral suppression for several years, which indicates that the infection is stable. However, aging is accompanied by multiple comorbidities, mainly high blood pressure and diabetes. This has been observed in all cohorts of OPLHIV aged 65 and over in the United States [24], France [9], Botswana [30], and South Africa [29]. The onset of chronic diseases combined with functional disabilities, such as reduced mobility and impairments in vision and hearing, complicates medical follow-up, increases healthcare costs, and directly affects daily life and independence.

The intersectional approach integrates biological and social aspects to provide an understanding of how different factors (biological, psychosocial, economic, gender-related, and cultural) interact with individual trajectories. Aging with HIV appears to be a unique experience shaped by an evolving set of factors, including gender, age, stigma, health status, marital status, the social integration of descendants, public policies, and the functioning of health systems [42]. These factors influence physical and mental health, emotional well-being, and access to care.

In 2024, social stigma associated with HIV remains a key determinant of how OPLHIV experience the disease in Senegal. This stigma is reinforced by cultural and religious factors as well as the low national prevalence of 0.3%, which renders PLHIV socially invisible. Although the trend is not uniform, studies in sub-Saharan Africa have shown a link between HIV prevalence and associated stigma [54]. In some contexts, higher HIV prevalence is associated with reduced stigma, suggesting that normalization of the disease could mitigate rejection [47].

In Senegal, a country with a low prevalence rate, fear of stigma persists despite decades of health information campaigns aimed at normalizing the disease and reducing negative stereotypes. Even community leaders are reluctant to speak out publicly in their own country, preferring to express themselves internationally.

Among older adults, these fears are amplified by the taboo surrounding the sexuality of older people. In many Western and African contexts, the social perception of older adults as asexual persists, perpetuating ageism and shame [5]. Older women face this issue twice over, as their sexuality is less tolerated than that of men. In “young” societies, such as those in sub-Saharan Africa where more than half of the population is under 19 years old [8], HIV is still perceived as a “disease of the young.” Older individuals diagnosed at an advanced age often feel ashamed and live in fear of disclosure, which could compromise their status and the respect associated with age [27].

The intersection of gender, age, stigma, and disease yields different outcomes [4]. Although women are disadvantaged by polygyny and socioeconomic conditions, they are often perceived as victims of infection and experience less stigma than men, particularly from their children. This enables them to receive support from their adult children and other social connections. For MSM, the situation is more complex. They are significantly more exposed to HIV than the rest of the population [53], and are persecuted because of their sexual orientation [2]. They face multiple stigmas and live in constant fear of disclosure. Their aging depends heavily on family and social circumstances, such as whether they have grateful children or nephews, whether they maintain or lose their economic and social capital after periods of exile, and the degree to which their sexual orientation is visible.

Beyond stigmatization, the main difficulties they encounter relate to chronic diseases and functional disabilities that impair quality of life and increase healthcare costs. Seeking care in facilities other than usual HIV centers creates additional stress related to cost, anonymity, and fear of involuntary disclosure.

Thus, the experience of aging with HIV differs depending on a combination of factors, including gender, sexual orientation, economic resources, access to social protection, and the presence of caregivers. These conditions are intertwined with the history of HIV status, which is often marked by biographical ruptures, as well as with the quality of social and family relationships built up over time. These constraints limit the recognition of OPLHIV in the political and social spheres. Invisible, they remain marginalized in social protection policies. Aging is not yet a priority for PLHIV associations, even though many of their leaders are over 60. Despite the experience these associations have gained in social and health support, specific initiatives remain rare.

## Public health perspective

From a public health perspective, analyzing the living conditions of OPLHIV in Senegal suggests possible interventions in various complementary areas [14,15].

First, health authorities must recognize the unique needs of PLHIV and implement targeted programs. The Senegalese health authorities have taken a step in this direction by devoting a chapter to the care of OPLHIV in the 2023-2030 National Strategic Plan for the first time [43].

Second, screening and care for age-related comorbidities and frailties must be integrated into HIV services. This integration requires developing geriatric skills, streamlining prescriptions, and promoting the use of lower-cost drugs and biological reagents. The project “VIHeillir: Aging Well with HIV in Cameroon and Senegal” is currently testing [41].

Furthermore, strengthening existing adapted health coverage mechanisms, such as the Sésame plan and mutual health insurance schemes, appears essential to reducing the economic vulnerability of OPLHIV. Similarly, developing social support measures in the form of a minimum old-age income would strengthen the economic autonomy of OPLHIV. With a view to generalizing this measure, it would also strengthen the economic autonomy of older people as a whole. Finally, strengthening the capacities of community actors appears essential. In Senegal, as in many African countries, community actors involved in HIV have extensive experience providing social support to PLHIV. They were able to mobilize this support remarkably in the collective response to the COVD-19 pandemic. This expertise could be built upon through initiatives such as the VIHeillir project, which mobilizes various patient associations and organizations of older people to define new forms of community support that strengthen the social inclusion of OPLHIV.

These interventions complement each other and should be implemented simultaneously to establish a foundation for strengthened health and social policies that promote inclusion and social equity for PLHIV.

## Conclusion

An analysis of the contemporary experience of aging with HIV in Senegal from the perspective of older people reveals several major elements with theoretical and practical implications.

Like all older adults in Senegal, OPLHIV experience the biological and social effects of aging. The onset of comorbidities and functional disabilities reduces their autonomy and increases their financial dependence on loved ones. The weakness of institutional social protection mechanisms and the absence of community support systems currently place the majority of the care burden on families.

HIV adds an additional level of vulnerability. The persistence of social stigma surrounding HIV continues to profoundly impact individuals’ lives. It leads OPLHIV to self-stigmatization and constant fear of the unexpected disclosure of their illness. For some, it leads to various forms of social self-exclusion. Maintaining secrecy shapes a significant part of their life and care trajectories. As they age and lose autonomy, they face economic difficulties that force them to seek help from those around them, which threatens to upset long-established psychosocial and relational balances. In the coming years, the issue of aging populations in Africa will require strengthening health and social policies (e.g., care for chronic diseases and social protection mechanisms) at the national level, as the public health implications of this issue are already apparent. At the heart of these policies, populations such as OPLHIV, who face multiple vulnerabilities, should receive specific treatment. The community, which has proven its effectiveness since the beginning of the fight against AIDS, will play a decisive role as both a whistleblower and a force for proposals and mobilization in favor of including OPLHIV.

## Ethical statement

Ethical and scientific opinion issued by the National Ethics Committee for Health Research in Senegal, No. 315/MSAS/CNERS/SP, Protocol SN21/96, version 2.0 of February 7, 2022, transmitted on October 16, 2023.

## Data availability

Open data on Dataverse: https://dataverse.ird.fr/dataset.xhtml?persistentId=doi: 10.23708/B09IQS

## Funding

Sidaction-Ensemble contre le sida, Paris, France; 2021

## Authors’ contributions

BT, GL-B, KS participated in the design of the research.

The field survey was conducted by SD, CF, MNN, PTS, KS, GLB, and BT, supervised by BT, GL-B, and KS.

The analyses were performed by BT, GL-B, KS, SD, CF, MNN, and PTS.

BT and GL-B drafted the first version of the document, which was critically reviewed and substantially expanded by KS.

All authors approved the final version.

## Conflicts of interest

The authors declare no conflicts of interest.
